# Profile of plasma microRNAs as a potential biomarker of Wilson’s disease

**DOI:** 10.1007/s00535-024-02135-6

**Published:** 2024-07-26

**Authors:** Ana Sánchez-Monteagudo, Edna Ripollés, Oihana Murillo, Sofia Domènech, María Álvarez-Sauco, Eva Girona, Isabel Sastre-Bataller, Ariadna Bono, Luis García-Villarreal, Antonio Tugores, Francisco García-García, Gloria González-Aseguinolaza, Marina Berenguer, Carmen Espinós

**Affiliations:** 1 Unit of Rare Neurodegenerative Diseases, Valencia Biomedical Research Foundation-Centro de Investigación Príncipe Felipe (CIPF), Calle Eduardo Primo Yúfera No. 13, 46012 Valencia, Spain; 2 Rare Diseases Joint Unit, CIPF-IIS La Fe, Valencia, Spain; 3 DNA@RNA Medicine Division, Centro de Investigación Médica Aplicada (CIMA), University of Navarra, Pamplona, Spain; 4 Department of Neurology, Hospital General Universitari d’Elx, Alicante, Spain; 5 Department of Internal Medicine, Hospital General Universitari d’Elx, Alicante, Spain; 6 Department of Neurology, Hospital Universitari i Politècnic La Fe, Valencia, Spain; 7 Hepatology-Liver Transplantation Unit, Digestive Medicine Service, IIS La Fe and CIBER-EHD, Hospital Universitari i Politècnic La Fe, Valencia, Spain; 8 Research Unit, Complejo Hospitalario Universitario Insular Materno-Infantil, Las Palmas de Gran Canaria, Spain; 9 Unit of Bioinformatics and Biostatistics, Valencia Biomedical Research Foundation-Centro de Investigación Príncipe Felipe (CIPF), Valencia, Spain; 10 Vivet Therapeutics S.L., Pamplona, Spain; 11 Department of Medicine, Universitat de València, Valencia, Spain; 12 Biotechnology Department, Universitat Politècnica de València, Valencia, Spain; 13 Rare Diseases Networking Biomedical Research Centre (CIBERER), Instituto de Salud Carlos III (ISCIII), Madrid, Spain

**Keywords:** miR-122-5p, miR-192-5p, miR-885-5p, Prognosis, Drug monitoring

## Abstract

**Background:**

Wilson’s disease (WD) is a rare condition resulting from autosomal recessive mutations in *ATP7B*, a copper transporter, manifesting with hepatic, neurological, and psychiatric symptoms. Timely diagnosis and appropriate treatment yield a positive prognosis, while delayed identification and/or insufficient therapy lead to a poor outcome. Our aim was to establish a prognostic method for WD by characterising biomarkers based on circulating microRNAs.

**Methods:**

We conducted investigations across three cohorts: discovery, validation (comprising unrelated patients), and follow-up (revisiting the discovery cohort 3 years later). All groups were compared to age- and gender-matched controls. Plasma microRNAs were analysed via RNA sequencing in the discovery cohort and subsequently validated using quantitative PCR in all three cohorts. To assess disease progression, we examined the microRNA profile in *Atp7b*^*−/−*^ mice, analysing serum samples from 6 to 44 weeks of age and liver samples at three time points: 20, 30, and 40 weeks of age.

**Results:**

In patients, elevated levels of the signature microRNAs (miR-122-5p, miR-192-5p, and miR-885-5p) correlated with serum activities of aspartate transaminase, alanine aminotransferase and gamma-glutamyl transferase. In *Atp7b*^*−/−*^ mice, levels of miR-122-5p and miR-192-5p (miR-885-5p lacking a murine orthologue) increased from 12 weeks of age in serum, while exhibiting fluctuations in the liver, possibly attributable to hepatocyte regenerative capacity post-injury and the release of hepatic microRNAs into the bloodstream.

**Conclusions:**

The upregulation of the signature miR-122-5p, miR-192-5p, and miR-885-5p in patients and their correlation with liver disease progression in WD mice support their potential as biomarkers of WD.

**Supplementary Information:**

The online version contains supplementary material available at 10.1007/s00535-024-02135-6.

## Introduction

Wilson’s disease (WD), a rare disorder (MIM 277900; ORPHA:905), with a prevalence ranging from 1/30,000 to 1/50,000 and a relatively high carrier frequency of 1/90, is characterised by autosomal recessive mutations in *ATP7B*, a copper transporter [1]. Improper functioning of ATP7B causes copper to accumulate in the liver, where it is released and enters the circulation. As a result, toxic copper accumulates in other organs, mainly in the brain, and also in the kidneys, eyes, heart, muscles, and bones [2]. Hence, the clinical presentation is diverse, with systemic damage stemming from copper accumulation in various tissues, particularly the liver and the brain. Liver damage can manifest as acute or chronic hepatitis, fulminant liver failure, or cirrhosis, with normal or elevated liver enzyme levels. Neurological symptoms may include parkinsonism, tremor, and dystonia, while psychiatric signs can involve behavioural changes and cognitive disorders. Fortunately, effective therapy exists, involving daily intake of copper chelators or zinc salts to promote urine copper excretion or reduce copper absorption, respectively, ultimately rebalancing copper levels. Early diagnosis and lifelong treatment adherence are crucial to prevent irreversible organ damage and disease progression.

Despite its significance, WD remains underdiagnosed. In France, the carrier frequency is 1/31, with a prevalence of around 3/200,000 [3]. Diagnosis relies on the Leipzig scale [4]. Incomplete penetrance, variable expressivity, and other disorders mimicking WD contribute to under diagnosis [5, 6]. Biochemical tests, like ceruloplasmin and copper assessments in blood, urine or liver, may be inconclusive [7].

Circulating microRNAs (miRNAs) have emerged as potential biomarkers for liver damage, serving as early indicators of disease and its progression in various pathologies, including metabolic dysfunction-associated steatotic liver disease (MASLD) and metabolic dysfunction-associated steatohepatitis (MASH) [8, 9]. MiRNAs regulate gene expression post-transcriptionally through mRNA degradation or translational repression [10, 11]. Notably, there is a strong correlation between circulating and tissue miRNAs in liver diseases [11].

This study explored plasma miRNAs as potential WD biomarkers. MiR-122-5p, miR-192-5p, and miR-885-5p exhibited altered levels in distinct patient cohorts. Findings were corroborated in an *Atp7b*^*−/−*^ mouse model [12–15] to study disease progression. Our findings support that detected miRNA signature may serve as a valuable biomarker for monitoring WD patients.

## Materials and methods

### Subjects

The research performed with human samples was approved by the Ethics Committee of the Hospital Universitari i Politècnic La Fe (Valencia, Spain; protocol code: 2019/0052; 22/05/2019) and was conducted in compliance with the principles of the Declaration of Helsinki, Good Clinical Practice guidelines, and local regulatory requirements. Informed consent was obtained from all participants prior to research initiation. All data were anonymised.

All of the experimental procedures involving animals were conducted in accordance with the European Union Guidelines for the Care (European Union Directive, 2010/63/EU) and the guidelines for the use of laboratory animals. The experimental design was approved by the Ethical Committee for Animal Testing of the University of Navarra (Pamplona, Spain; protocol code: CEEA/066-22; 02/09/2022).

Statistical power of the data was evaluated from the normalised counts of the experimental subjects, using Bioconductor’s RnaSeqSampleSize R package (https://www.bioconductor.org/packages/release/bioc/html/RnaSeqSampleSize.html). For a cohort of 20 individuals, a probability (power) of 0.70 was obtained, considering that 10% of miRNAs with a differential expression profile may have a relevant role.

#### Patients

A first cohort (discovery) including 20 in-depth phenotyped WD patients (Supplementary Table S1) was screened by high-throughput small RNA-seq (RNA-sequencing). For validation purposes, a second cohort (validation) and a third cohort (follow-up) comprising 22 and 25 WD patients, respectively (Supplementary Tables S1 and S2), were screened by quantitative PCR (qPCR). The discovery and follow-up cohorts comprised the same patients studied 3 years apart. Two patients included in the discovery cohort could not be further studied because they did not attend the medical check-up, while seven new patients diagnosed during these years and not evaluated in the discovery cohort were included in the follow up cohort. Individuals from the discovery and follow-up cohorts were supervised at Hospital Universitari i Politècnic La Fe (Valencia, Spain) and at Hospital General Universitari d’Elx (Alicante, Spain). Individuals from the validation cohort were recruited at Complejo Hospitalario Universitario Insular Materno Infantil de Gran Canaria (Las Palmas, Spain). These clinical series have been partially reported [6, 16]. Differential diagnosis is based on clinical features, biochemical criteria, presence of corneal Kaiser-Fleischer (KF) ring and other hepatic disturbances. All patients included met the following inclusion criteria: (1) Leipzig score ≥ 3 at diagnosis (without considering *ATP7B* mutations); and (2) conclusive genetic analysis. Other chronic liver diseases (hepatitis C or B, immune mediated liver diseases or alcohol related liver disease) were ruled out. We excluded patients who had undergone liver transplantation. Supplementary Tables S1 and S2 show the clinical and biochemical features of the patients.

Peripheral blood samples for plasma isolation and biochemical analysis were obtained during clinical follow-up visits. Biochemical determinations analysed using standard protocols (upon request): AST, aspartate transaminase; ALT, alanine aminotransferase; GGT, gamma-glutamyl transferase; bilirubin; AP, alkaline phosphatase; cholesterol; and triglycerides. For miRNA analyses, EDTA-blood samples were processed for plasma separation within 2–3 h following collection. After centrifugation at 1900×*g* for 10 min (4 °C), plasma fraction was aliquoted and stored at – 80 °C.

#### Healthy subjects

Plasma from controls were provided by the Biobanco para la Investigación Biomédica y en Salud Pública de la Comunitat Valenciana (PT13/0010/0064), integrated in the Spanish National Biobanks Network and in the Valencian Biobanking Network. Samples were processed following standard operating procedures. Demographic data from controls recorded in general health and lifestyle questionnaires were provided (Supplementary Table S3). Healthy subjects were matched by sex and age with patients, and those who registered neoplasms, cardiovascular, respiratory, bone, mental or endocrine disease risk factors were discarded.

#### Mice

Serum and liver samples of wild-type (WT) and *Atp7b*^*−/−*^ (WD) mice were received from Dra. González-Aseguinolaza laboratory (Centro de Investigación Médica Aplicada, Pamplona, Spain). *Atp7b*^*−/−*^ on a C57BL/6 J background were bred and maintained under pathogen-free conditions and genotyped at 3 weeks of age as described [14, 15]. These *Atp7b*^*−/−*^ mice show no ATP7B expression in the liver and exhibit the typical biochemical and physiopathological alterations observed in WD patients, except for the neurological signs [12, 13]. Alterations comprise high copper excretion in urine, low holoceruloplasminemia, high serum transaminase levels and increased liver copper concentration with associated hepatocellular damage [12–15].

The cohort comprised 20 or 10 WD mice for the studies using serum or liver, respectively, with the same number of WT mice. Serum samples were collected at 6–7, 10, 12, 16, 20, 30 and 40 weeks old, while liver samples were obtained at 20, 30 and 40 weeks old. Samples were immediately frozen at -80ºC and no tissue preservatives were used.

### Circulating miRNA-enriched total RNA isolation

Total RNA enriched for circulating miRNA fraction was extracted from 400 µL human plasma, 10 µL mouse serum and 20 mg mouse liver per subject and column using miRNeasy Mini kit (Qiagen, Hilden, Germany), following manufacturer’s protocol (miRNeasy Mini Handbook 11/2020). Aliquots from plasma/serum samples were thawed on ice and centrifuged (16,000×*g*, 5 min, 4 °C) to remove cell debris prior to RNA isolation. During isolation from mouse serum, 5.1 × 10^8^ copies of synthetic cel-miR-39 (IDT, Leuven, Belgium) were added into each sample to be used as exogenous miRNA reference.

### Small RNA library preparation and high-throughput sequencing

Individual small RNA libraries were prepared from 6 µL of total RNA enriched for circulating miRNA fraction isolated from human plasma samples with NEBNext Multiplex Small RNA Library Prep kit (New England Biolabs, Ipswich, MA, USA). To improve final miRNA sequencing yield, library prep protocol was optimised according to dilution 1:5 of 3′ and 5′ SR RT primer and 16× PCR cycles for indexing. Pre- and post-gel size selection PCR-indexed libraries were purified with Nucleospin Gel and PCR Clean-Up (Macherey–Nagel, Düren, Germany) following manufacturer’s protocol (Macherey–Nagel—03/2023, Rev. 08) for PCR clean-up and DNA purification from polyacrylamide gel, respectively.

Pre- and post-gel size selection library QC (Quality Control) was performed using High Sensitivity D1000 ScreenTape in a TapeStation 4200 (Agilent Technologies, Santa Clara, CA, USA). Post-gel purified libraries were quantified by qPCR with KAPA Library Quantification Kit (Kapa Biosystems, Wilmington, MA, USA) and pooled for sequencing in Illumina HiSeq 2500 (1 × 50 bp, v4).

### Bioinformatics analyses

#### Primary analysis

FASTQ raw data files obtained from small RNA-seq were processed for computational analysis. Before and after removing adapter sequences and selecting reads by size with Cutadapt (v2.6), QC with FastQC (v0.11.8) was performed to check if reads size distribution and quantity was compatible with mature miRNA (16–28 bp) and sufficient for differential representation analyses. Selected reads were aligned against pre-miRNA sequences (hairpin) from the latest miRBase v22 using Bowtie (v1.1.2). Counts of reads matching hairpin arms were obtained with Subread (v1.6.0) and a custom GFF (General Feature Format) file with 5p and 3p coordinates corresponding to annotated human mature miRNAs in miRBase v22.

#### Differential expression analysis and functional enrichment

To identify differential expressed mature miRNAs, edgeR package was used [17]. Starting from extracted mature miRNA counts, those low-represented were filtered before setting per-sample library size for normalisation by the trimmed mean of M values (TMM) method. QLF (Quasi-Likelihood F) and LRT (Likelihood Ratio Test), both considered generalised linear models (GLM), were applied to determine differentially expressed mature miRNAs in patients compared to controls (design 1), and in a second approach, including the covariables sex and age, to adjust comparisons with controls (design 2). miRNAs with a false discovery rate (FDR) < 0.05 were considered as significantly deregulated.

### miRNA detection by quantitative PCR

To obtain cDNA from mature miRNAs, a 2–2.5 µL total RNA enriched for circulating miRNA fraction isolated from human plasma or murine serum or 10 ng if isolated from tissue sample was used with the TaqMan Advanced miRNA cDNA Synthesis Kit (Applied Biosystems, Foster City, CA, USA).

Levels of mature miRNAs of interest were detected by qPCR with TaqMan Advanced (Applied Biosystems, Foster City, CA, USA) probes (Supplementary Table S4) and master mix in a LightCycler 480 II (Roche, Mannheim, Germany) programmed. Pre-amplified cDNA was diluted 1/10 and 2.5 µL per reaction were used as template for qPCR, scaling reaction components to a 10 µL final volume.

Relative miRNA expression levels in case sample (human or murine) compared to control sample was calculated as ${2}^{-\left(\Delta \text{CtCase}-\text{Average}\Delta \text{CtControl}\right)}$, being $\Delta Ct={\text{Ct}}_{\text{targetmiRNA}}-{\text{AverageCt}}_{\text{referencemiRNA}}$. As reference miRNAs for qPCR analysis in human plasma samples, endogenous levels of miR-16-5p and miR-484 were used, while for mouse serum, endogenous miR-484 and exogenous cel-miR-39 spike-in levels were considered to normalise data. Finally, for mouse liver samples, endogenous miR-16-5p was used as reference miRNA.

### Statistical analyses

Shapiro–Wilk test was applied to biochemical parameters to check for normality distribution, and as all resulted negative, non-parametric Wilcoxon signed-rank test was used. Paired *t* test was performed to assess differences of relative miRNAs expression levels, represented as log_2_FC, between cases and controls. For in-group comparisons, two-sample *t* test was used instead.

For murine samples, Shapiro–Wilk and Levene tests were applied to check for normality distribution and homoscedasticity of miRNA expression levels represented as log_2_FC. Two-way mixed ANOVA (analysis of variance between-sex factor and within-age factor) and pairwise post hoc *t* test were used to determine differences between groups.

The Spearman’s Rho (*R*_s_) coefficient of correlation was used to establish the association of relative miRNAs expression levels with biochemical parameters in human plasma and in murine serum, and additionally, with Leipzig score (including *ATP7B* mutations) and age in human plasma.

## Results

### Cohorts

Studies were first performed in the discovery cohort by small RNA-seq, and they were further validated in both cohorts, discovery and validation, by qPCR. Supplementary Table S5 shows the demographic and clinical characteristics of the patient cohorts. Both cohorts are quite similar regarding the number of women *versus* men, with mean ages between 36 and 43 years old. The hepatic presentation was more frequent than the neurologic one, whereas the mixed presentation was rare. Asymptomatic individuals were detected in mutational screenings of relatives. Biochemical determinations resulted to be quite homogeneous between both cohorts, and although ALT levels were more disparate, differences among groups were not significant (Supplementary Table S5).

### Determination of miRNAs in plasma of WD patients and controls

Initially, we assessed the quantity and quality of reads obtained in small miRNA-seq, focusing on their processing. Across the 40 sequenced samples, approximately 4–8 million raw reads were obtained, reduced to 4–6 million after removing library adapters and discarding reads smaller than 15 bp (Supplementary Figure S1). Subsequently, 2–4 million reads per sample, corresponding to mature miRNAs (size 16–28 bp), were selected for alignment against precursor miRNA sequences from miRBase v.22. For differential representation analysis, counts of sequences mapped to the determined coordinates in the 5p and 3p arms of each precursor, representing mature miRNAs, were obtained. Proportionally, the number of mapped readings belonging to mature miRNAs sequences was higher in the patient group, reflecting the physiological differences between the two groups (Supplementary Figure S1). After filtering and normalisation, 2158 mature miRNAs out of 2650 were retained, excluding low-represented miRNAs.

Two different experimental designs were applied for differential representation analysis: design 2 considers as covariables sex and age (Supplementary Figure S2). The LRT method identified 42 and 37 differentially expressed miRNAs between designs 1 and 2, respectively (Supplementary Figure S2). The QLF method revealed 29 deregulated miRNAs in design 1 and 30 in design 2. Importantly, 18 miRNAs were common for the four strategies, and therefore, do not depend on the covariates age and sex. The 18 miRNAs presented a positive log_2_FC value, and hence, they would be overrepresented in patient plasmas. Supplementary Table S6 lists the results obtained using both methods, QLF and LRT, with the two designs.

### Profile of miRNAs and correlation with biochemical parameters

From the list of 18 miRNAs, eight miRNAs were selected for further validation (Supplementary Table S6). Seven miRNAs (miR-122-5p, miR-193b-5p, miR-885-5p, miR-885-3p, miR-455-5p, miR-485-3p and miR-340-3p) were selected based on the following criteria: (1) value of log_2_FC > 1.4 in the differential representation analysis in both experimental designs; and (2) association with liver disease and/or metal metabolism. Of note, because miR-122b-5p, miR-122b-3p and miR-122-5p belong to the same family, we only included miR-122-5p, which is the most studied isoform related to hepatic disease. In addition, the miR-192-5p was also investigated because this miRNA is the second most abundant miRNA in the liver (after miR-122) and is widely related to liver damage [18].

**Fig. 1:**
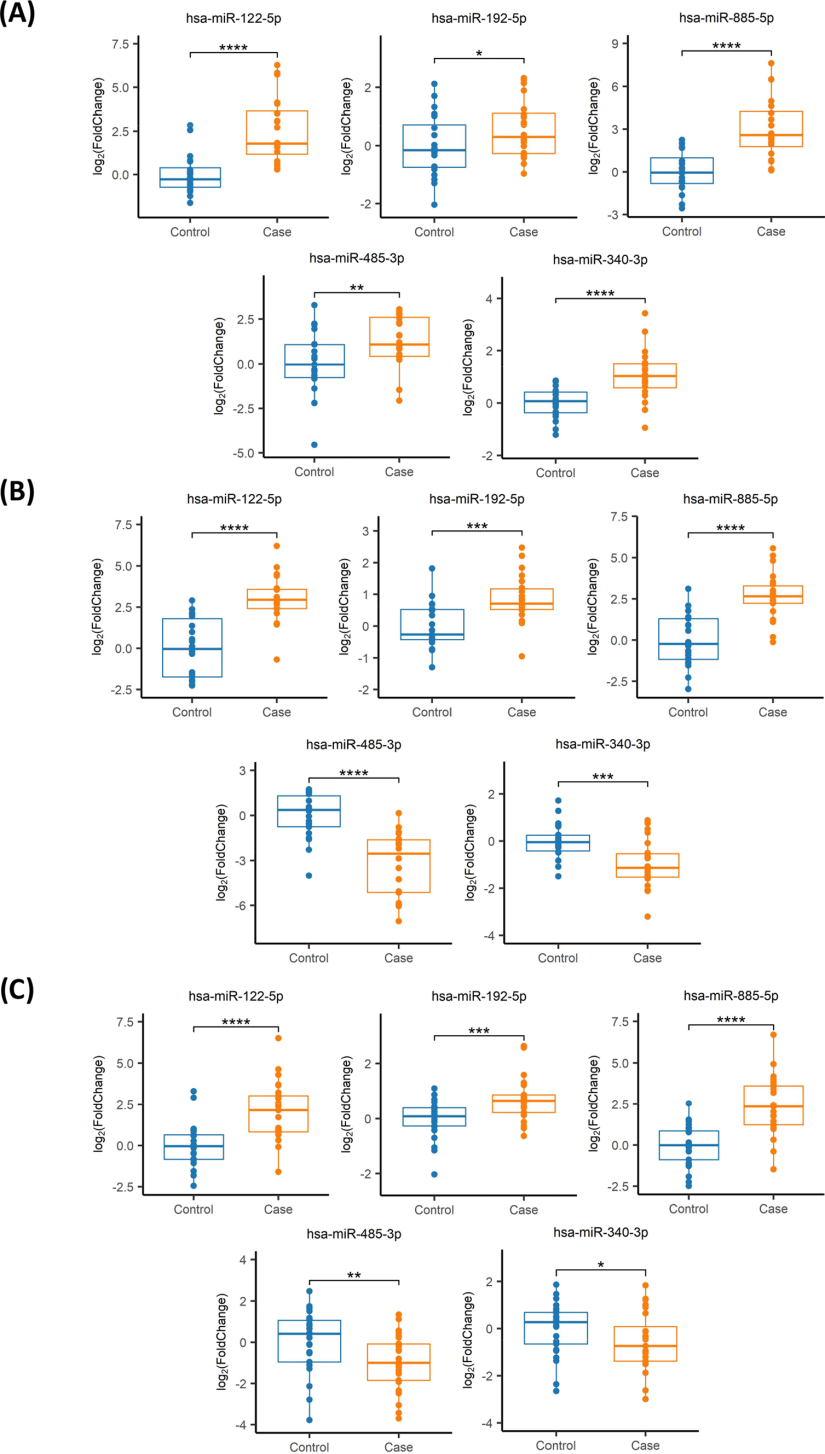
Analysis of the miRNA profile. **a** Discovery, **b** validation, and **c** follow-up cohorts. Comparative analysis of miRNA levels between patients and controls, representing IQR (interquartile range) of log_2_FC relative expression (2^−ΔΔCt^). ****P value < 0.0001; ***P value < 0.001; **P value < 0.01; *P value < 0.05

**Fig. 2:**
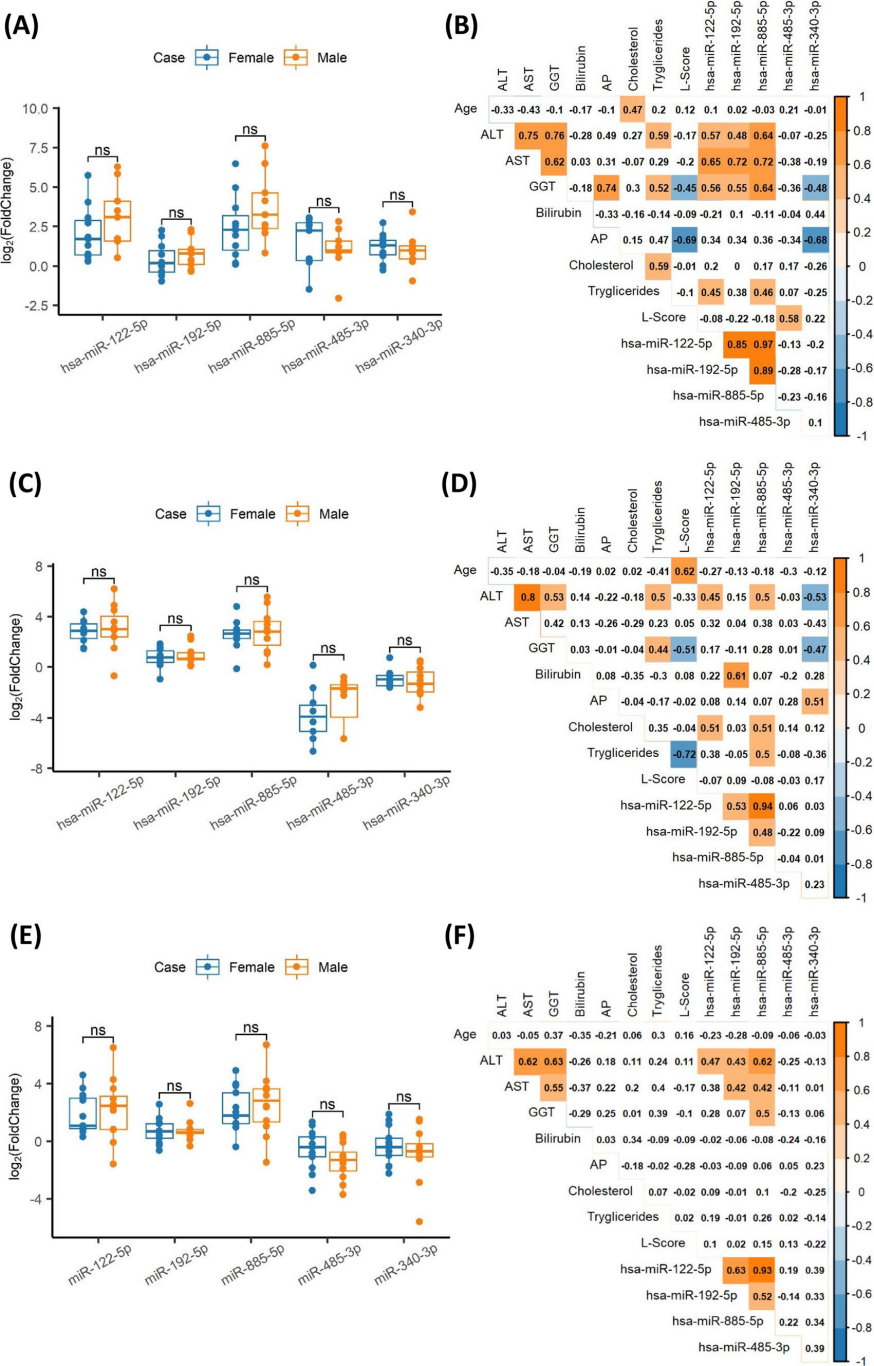
Comparative analysis of the relative expression of miRNA levels by gender from case groups from the **a** discovery, **c** validation, and **e** follow-up cohorts. Correlation matrix including biochemical parameters, age, and levels of miRNAs profile in patients belonging to the **b** discovery, **d** validation, and **f** follow-up cohorts. In each cell, Spearman’s coefficient (*R*_s_) of correlation by pair of variables is indicated. Coloured cells represent significant correlation (*P value < 0.05) in orange when positive, and in blue when negative. *ns* not significant, *AP* alkaline phosphatase, *AST* aspartate aminotransferase, *ALT* alanine aminotransferase, *GGT* gamma-glutamyl transferase, *L-score* Leipzig score

Three (miR-193b-5p, miR-455-5p and miR-885-3p) out of the eight selected miRNAs were ruled out because they had an indistinguishable fluorescence signal. Therefore, five miRNAs were validated by qPCR in the discovery cohort (Supplementary Table S6, shaded in blue). Levels for miR-122-5p, miR-192-5p, miR-885-5p, miR-485-3p, and miR-340-3p were significantly higher in the patients (Fig. 1a), consistent with RNA-seq findings. Using the same approach in the validation (Fig. 1b) and follow-up cohorts (Fig. 1c), miR-122-5p, miR-192-5p, and miR-885-5p exhibited consistently increased levels in patient plasmas. In contrast, levels of miR-485-3p and miR-340-3p were significantly decreased (Fig. 1b, c), leading to inconclusive findings. Finally, no significant differences were observed when cohorts were split by sex (Fig. 2a, c, e), and age did not influence miRNA levels in plasma across the studied cohorts (Fig. 2b, d, f).

Positive correlations were detected between AST, ALT, and GGT and miR-122-5p, miR-192-5p, and miR-885-5p (Fig. 2b, d, f), supporting their association with liver disease. Triglycerides and/or cholesterol showed a positive correlation with miR-122-5p and miR-885-5p. Additionally, miR-192-5p showed a positive and significant correlation with total bilirubin, although only in the validation cohort. Notably, *R*_s_ coefficients between miR-122-5p, miR-192-5p, and miR-885-5p were consistently significant across all cohorts (*R*_s_: 0.48–0.97) (Fig. 2b, d, f), emphasizing their participation in common pathways. Based on all the above findings, the miRNA profile -miR-122-5p, miR-192-5p, and miR-885-5p- emerged as a potential biomarker for WD.

MiR-485-3p did not show significant correlations in any cohort (Fig. 2b, d, f) while miR-340-3p displayed variable correlations with biochemical parameters (Fig. 2b, d). Additionally, neither miR-485-3p nor miR-340-3p exhibited notable associations with the other three miRNAs across the cohorts. Therefore, miR-485-3p and miR-340-3p presented conflicting findings, hindering a definitive conclusion regarding their role as WD biomarkers.

Significant correlation with the Leipzig score was only detected for miR-485-3p in the discovery cohort, and was not replicated in the remaining cohorts of patients (Fig. 2b, d, f).

### Profile of circulating miRNAs in serum and liver of a mouse model of WD

**Fig. 3:**
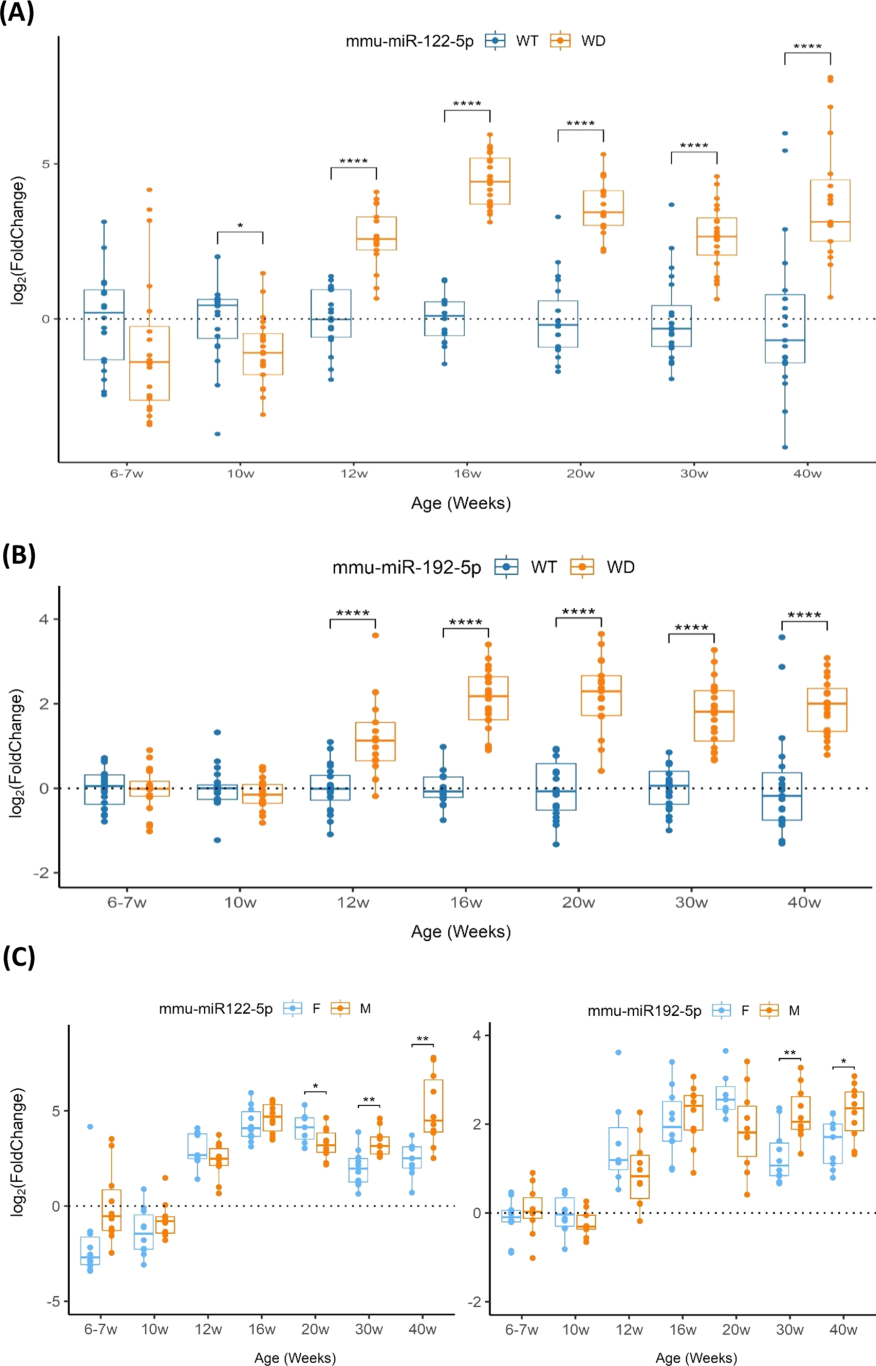
Validation of the miRNAs profile in serum of wild-type (WT) and *Atp7b*^*−/−*^ mice (WD, Wilson’s disease) samples. **a** Comparative analysis of miR-122 levels and, **b** miR-192 levels between groups at different ages, representing IQR (interquartile range) of log_2_FC relative expression (2^−ΔΔCt^). **c** Comparative analysis of relative expression miRNA levels from *Atp7b*^*−/−*^ group from **a** and **b** by sex. ****P value 0.0001; **P value < 0.01; *P value < 0.05

The miRNA profile in the *Atp7b*^*−/−*^ mice [12–15] was investigated to establish the presentation pattern as the disease progresses. Unfortunately, miR-885-5p lacks its murine orthologue. Since we used human plasma, we first tested miR-122-5p and miR-192-5p in plasma from five WT and WD mice at the age of 21 weeks. Both miRNAs were significantly overrepresented in the WD mice (data not shown). Next, we investigated both miRNAs in serum from WT and WD mice. Significantly increased levels of miR-122-5p and miR-192-5p were detected from the age of 12 weeks to 40 (Fig. 3a, b).

We investigated the influence of sex in the presentation of the miRNAs in serum in the *Atp7b*^*−/−*^ mice. Significant differences were observed at the ages of 30 and 40 weeks (Fig. 3c), when disease is in advanced state: miR-122-5p and miR-192-5p had an increased presentation in males compared to females.

In WD mice, we found a positive R_s_ coefficient of the miRNA presentation with ALT and AST (GGT was not available in mice) starting at 16 weeks, with correlations being significant in most estimates (Supplementary Figure S3). In addition, the *R*_s_ coefficient between miR-122-5p and miR-192-5p showed a strong association (*R*_s_ > 0.58) in all ages.

**Fig. 4:**
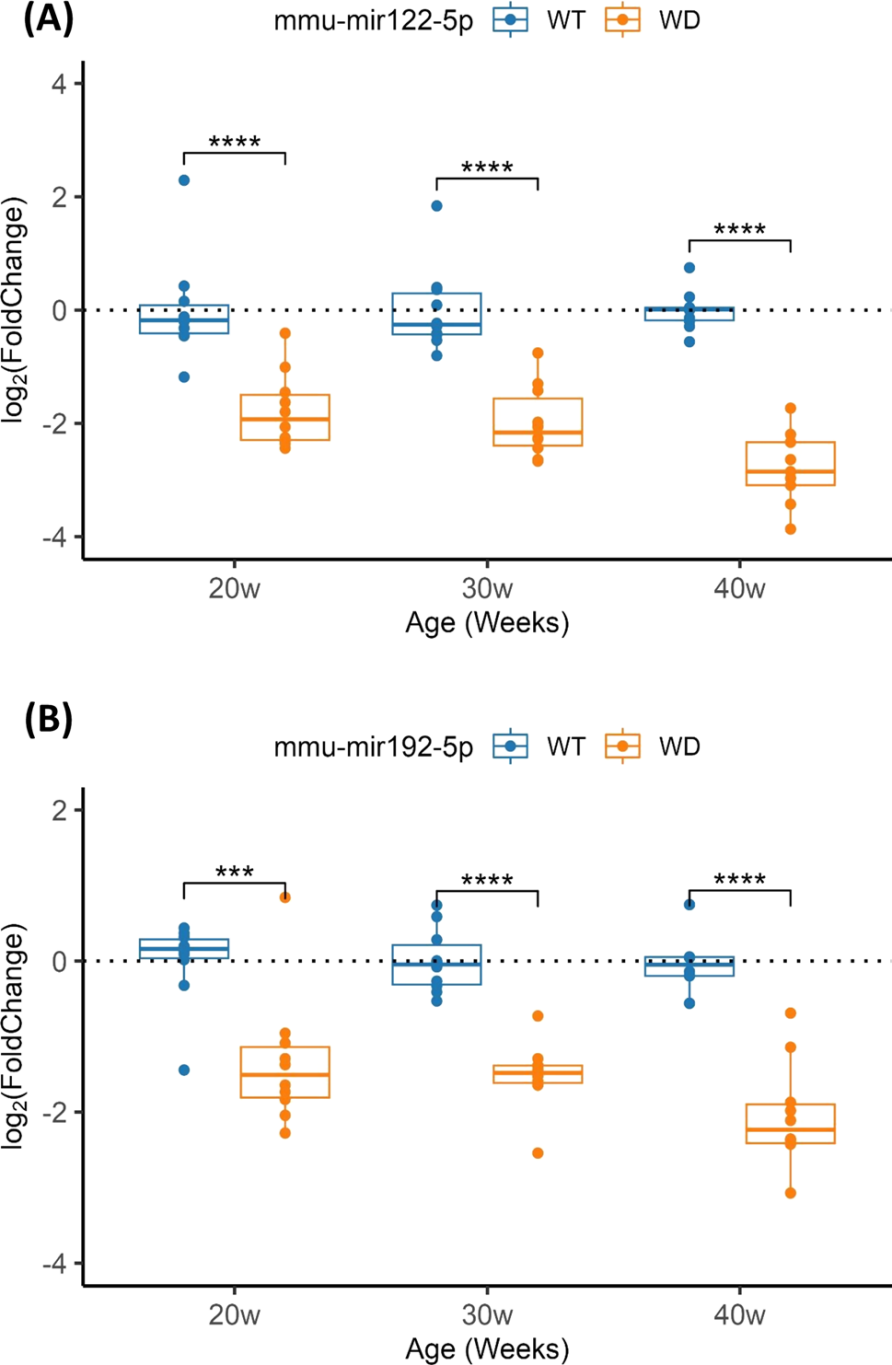
Validation of the liver tissue miRNAs profile in wild-type (WT) and *Atp7b*^*−/−*^ (WD) mice samples. **a** Comparative analysis of miR-122 levels and, **b** miR-192 levels between groups at different ages, representing IQR (interquartile range) of log_2_FC relative expression (2^−ΔΔCt^)

Lastly, we investigated miR-122-5p and miR-192-5p in liver from mice at 20, 30 and 40 weeks. Both miRNAs presented significantly decreased levels compared to WT, mainly at 40 weeks of age (Fig. 4).

## Discussion

Our objective was to assess the utility of plasma miRNAs as biomarkers of WD patients. In human plasma across three patient cohorts, miR-122-5p, miR-192-5p, and miR-885-5p displayed significantly elevated levels compared to healthy controls. In a *Atp7b*^*−/−*^ mouse model [12–15], miR-122-5p and miR-192-5p in serum mirrored the pattern observed in patients, while liver levels of both miRNAs decreased, especially at 40 weeks, coinciding with the progression from chronic hepatitis (30 weeks) to cirrhosis (36–44 weeks) [14]. The fluctuation in liver miRNA levels could be attributed partly to hepatocyte regenerative capacity and the release of miRNAs into circulation during disease progression [19].

MiRNAs from the liver may enter the bloodstream passively through apoptosis and necrosis or actively through exosome and viral particle secretion [20]. Therefore, hepatic miRNA levels correlate with serum/plasma levels, offering a non-intrusive method to study liver injury. MiR-122 is associated with hepatocyte differentiation, development, and homeostasis [11]. Inhibition of miR-122 leads to inflammation, necrosis, steatosis and fibrosis [21]. Circulating miRNA-122 is used as biomarker of liver injury in chronic hepatitis B or C, hepatocellular carcinoma, MASH / MASLD, drug-induced liver disease (DILI) and alcohol-related liver disease (ALD) [8, 22–24]. In Long-Evans Cinnamon (LEC) rats used as WD models after induction of fulminant hepatitis with a high copper diet, levels of miR-122 in serum resulted to be significantly increased before the elevation of hepatitis-associated serum markers ALT, AST and bilirubin [19]. Similarly, in a rat model of MASLD, miR-122 was also superior to clinical markers traditionally used to monitor hepatic disease [25]. Moreover, in a study encompassing liver diseases of different aetiology, circulating miR-122 was proposed as a disease severity-dependent biomarker at an early stage because its levels became elevated earlier than that of serum ALT [26]. In the WD model here used [12–15], there was a substantial increase in levels of miRNA-122-5p as well as miRNA-192-5p at week 12 (*P* value < 0.0001).

The *R*_s_ coefficients of miR-122-5p, miR-192-5p, and miR-885-5p demonstrated a significant and strong association, indicating their involvement in common pathways. These miRNAs are frequently found in profiles associated with liver injury [27–31]. Higher serum levels of a miRNA signature (which included miR-885-5p, miR-122-5p and miR-192-5p) have been found to be upregulated in chronic hepatitis B and MASLD [28, 29]. Correlation with ALT, AST and GGT was found for miR-885-5p and miR-122, and even a role regulating ALT activity was proposed for miR-122 [30, 31]. In line with this, our study identified a positive association between the miRNA signature and liver enzymes (ALT, AST, and GGT).

Significant correlation between miRNAs and the Leipzig score was only detected for miR-485-3p in the discovery cohort, discarded as a potential biomarker for WD. The absence of correlation between miRNAs and the Leipzig score could, therefore, be due to different type of data to be considered clinical signs, histopathological studies, biochemical tests and genetic analysis [4].

A role of sex was found in a study with a stronger association in men between the miR-122-5p and miR-885-5p and steatotic liver [31]. In addition, sex differences observed in the *Atp7b*^*−/−*^ mice at 30–40 weeks of age, when the liver is cirrhotic, were described to be associated with higher survival rates in females [15]. In our study, no differences were appreciated between women and men, suggesting the need for further exploration in larger patient series.

In conclusion, the increased levels of the miRNA profile miR-122-5p, miR-192-5p and miR-885-5p in plasma of WD patients compared to healthy controls and its association with liver enzymes suggest it might be a potential non-invasive biomarker in this disease. Some of these miRNAs (isolated or with others) have also been reported to be upregulated in other hepatic conditions. A further step would be to assess the role of this miRNA profile as a biomarker determining treatment response. While we did not study this, sensitive biomarkers, such as circulating miRNAs, hold promise for both prognosis of liver disease and assessment of treatment response, vital for identifying optimal therapeutic strategies.

## Supplementary Information

Below is the link to the electronic supplementary material.

Supplementary file1 (PDF 721 KB)

Supplementary file2 (DOCX 66 KB)
