# Supplementary material for: Profile of plasma microRNAs as a potential biomarker of Wilson’s disease
Source: J Gastroenterol. 2024 Jul 26;59(10):921–31. doi: 10.1007/s00535-024-02135-6 (PMC11415402; doi:10.1007/s00535-024-02135-6)
Supplement: Supplementary file 1 — Supplementary file1 (PDF 721 KB) [file 535_2024_2135_MOESM1_ESM.pdf]

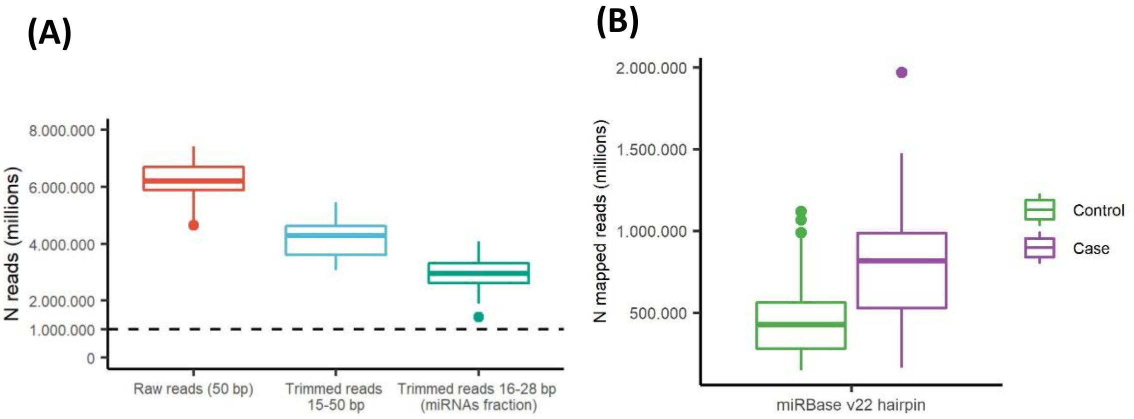

Figure S1 Quality control (QC) of mapped reads obtained in miRNA-seq of the discovery cohort. **a** Raw and trimmed reads distribution and size selection. **b** Distribution of reads (16-28 bp) aligned to hairpin 5p and 3p arms annotated in miRBase v.22.

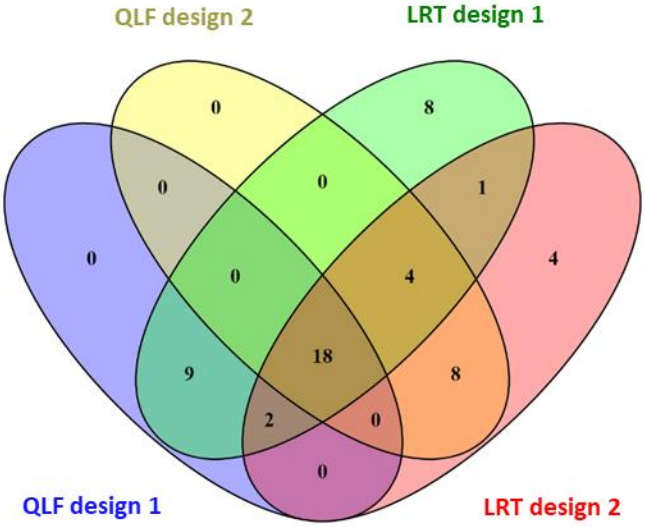

Figure S2 Concordance of mature miRNAs according the four strategies of analysis of differential representation. QLF (quasi-likelihood F) and LRT (Likehood ratio test) were applied to determine differentially expressed mature miRNAs in patients compared to controls (design 1), and in a second approach, including the covariables sex and age, to adjust comparisons with controls (design 2). miRNAs with FDR (false discovery rate)<0.05 were identified as significantly dysregulated.

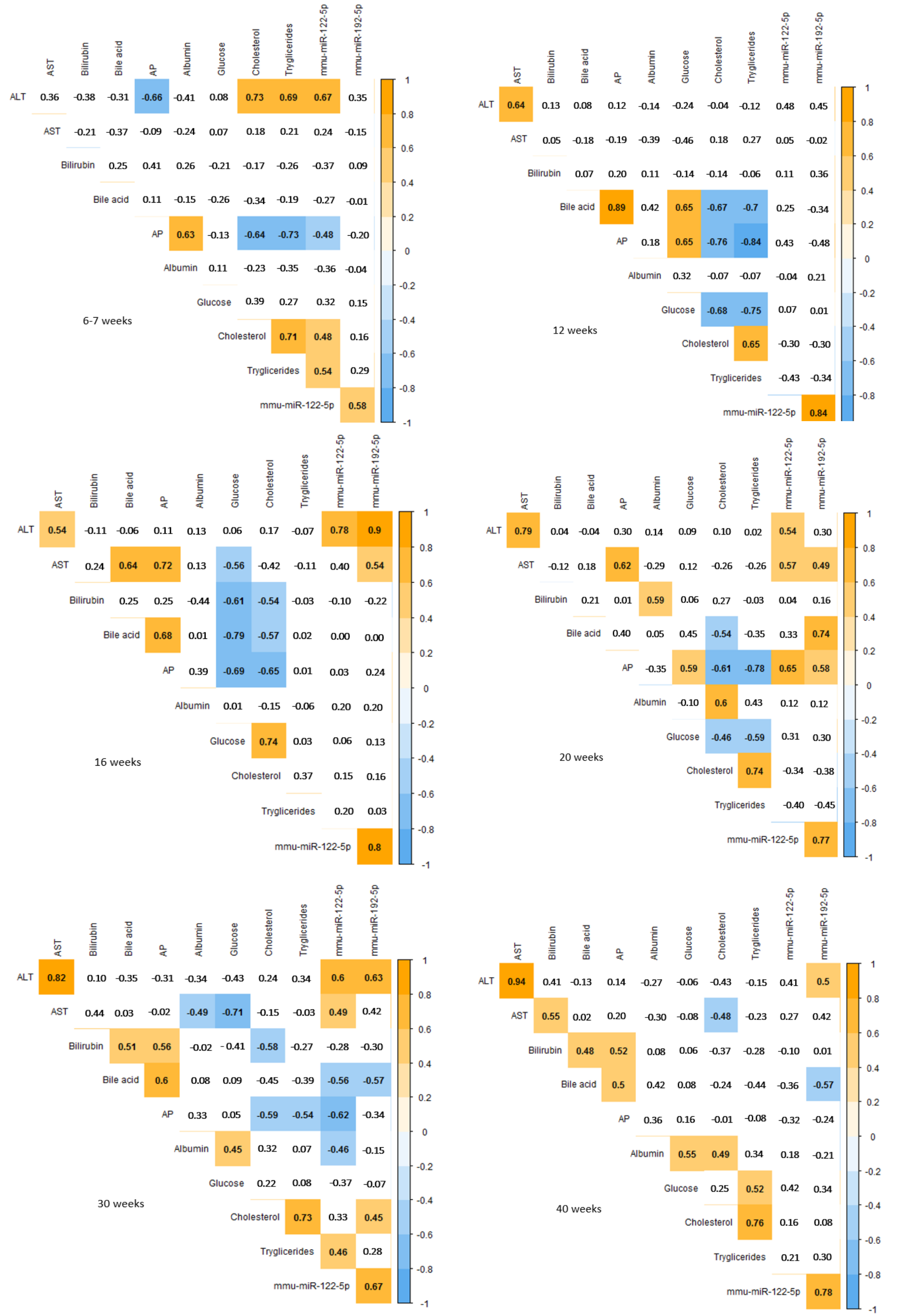

Figure S3 Correlation matrix including biochemical parameters and levels of serum circulating miRNAs profile from *Atp7b*$^{-/-}$ group at different age (6 to 40 weeks). In each cell, Spearman's coefficient of correlation (rho) by pair of variables is indicated. Coloured cells represent significant correlation (**P*-value<0.05) of positive value (in orange) or negative (in blue).
