# Supplementary material for: Profile of plasma microRNAs as a potential biomarker of Wilson’s disease
Source: J Gastroenterol. 2024 Jul 26;59(10):921–31. doi: 10.1007/s00535-024-02135-6 (PMC11415402; doi:10.1007/s00535-024-02135-6)
Supplement: Supplementary file 2 — Supplementary file2 (DOCX 66 KB) [file 535_2024_2135_MOESM2_ESM.docx]

**Table S1** Clinical features of the Discovery and the Follow-up Cohorts.

| Family | *ATP7B* mutations | Patient | Sex | Age (years) | | Presentation | KF  ring | CP^a^ | Copper | | | Treatment | Outcome | Leipzig  score^e^ |
| --- | --- | --- | --- | --- | --- | --- | --- | --- | --- | --- | --- | --- | --- | --- |
|  |  |  |  | **current** | **DG** |  |  |  | **Liver^b^** | **Serum^c^** | **24h Urine^d^** |  |  |  |
| fEW14 | p.M645R /  p.T977M | EW20^f^ | W | 31 | 7 | Hypertransaminasemia | - | 6 | NA | 26 | 507 | ZnAc | Biochemical remission | 4 |
| fEW15 | p.G1341V /  c.(?_-1627)_(51+1_52-1)del | EW24^f^ | W | 44 | 23 | Fibrosis (F4),  Hypertransaminasemia | + | 3 | 1200 | 22 | NA | ZnAc | Biochemical remission | 6 |
| fEW16 | p.A1135Qfs*13  (homozygous) | EW25 | W | 44 | 13 | Steatosis, hypertransaminasemia, hypercholesterolemia | - | 5 | NA | 27 | NA | ZnAc | Biochemical remission | 3 |
| fEW17 | p.Q111* /  p.L1120* | EW26^g^ | M | 36 | 10 | Hypertransaminasemia. | - | 2 | 564 | 17 | NA | ZnAc | No biochemical remission | 6 |
|  |  | EW44^g^ | M | 37 | 10 | Hypertransaminasemia. | - | 7 | 593 | 18 | NA | ZnAc | No biochemical remission | 6 |
| fEW19 | p.Q111* /  p.R778G | EW28 | M | 21 | 7 | Fibrosis (F2-3), hypertransaminasemia | + | 6 | 244 | 17 | 45 | ZnAc,  trientine | Biochemical remission | 3 |
| fEW20 | p.M645R /  c.4124+5G>A | EW29^g^ | M | 23 | 6 | Hypertransaminasemia | - | 2 | 864 | 23 | 123 | D-Pen +  ZnAc | Biochemical remission | 4 |
| fEW29 | p.M645R /  c.2447+1G>T | EW45 | M | 34 | 7 | Fibrosis (F2-3), hypertransaminasemia | - | 5 | 405 | 39 | 476 | D-Pen +  vitamin B_6_ | Biochemical remission | 6 |
| fEW30 | p.H580Pfs*2 /  p.H1069Q | EW46 | W | 48 | 22 | Fibrosis (F3-4).  Dystonia, postural tremor, dysarthria, dystonic gait, psychiatric symptoms | + | 2 | NA | 17 | NA | ZnAc | Neurological improvement | 5 |
| fEW31 | p.M645R /  c.4125-1G>C | EW49 | W | 45 | 17 | Hypertransaminasemia, hypercholesterolemia | - | 7 | 600 | 37 | NA | ZnAc | Biochemical remission | 6 |
| fEW33 | c.1708-1G>A  (homozygous) | EW54 | M | 48 | 35 | Fibrosis (F3-4), hypertransaminasemia, hypercholesterolemia. Parkinsonian syndrome | + | 17 | NA | 86 | 598 | ZnAc | Neurological improvement | 4 |
| fEW36 | p.I1311T /  p.G1086Rfs*32+p.L1120* | EW58 | M | 39 | 33 | Hypertransaminasemia. Tremor, dystonia, dysarthria | + | 0 | NA | < 20 | ND | ZnAc | No neurological improvement | 8 |
| fEW37 | c.1708-1G>A  (homozygous) | EW62 | M | 32 | 18 | Hypertransaminasemia.  Dystonia, psychiatric symptoms | + | 12 | NA | 46 | 1129 | ZnAc | No neurological improvement | 7 |
| fEW38 | p.M645R /  p.R778W | EW65 | M | 56 | 48 | Cirrhosis, hypertransaminasemia | - | 8 | NA | 40 | NA | ZnAc | Biochemical remission | 4 |
| fEW39 | p.R778G /  p.N1324S | EW66 | W | 39 | 15 | Cirrhosis | - | 6 | NA | NA | NA | ZnAc | Asymptomatic | 3 |
|  |  | EW67 | M | 30 | 7 | Hypertransaminasemia | - | NA | NA | NA | 147 | D-Pen | Asymptomatic | 3 |
|  |  | EW68 | W | 34 | 9 | Hypertransaminasemia | - | NA | NA | NA | 143 | D-Pen | Asymptomatic | 3 |
|  |  | EW69 | W | 29 | 5 | Mild hypertransaminasemia | - | 4 | NA | NA | 155 | D-Pen | No biochemical remission | 3 |
| fEW40 | c.51+4A>T /  p.L1120* | EW70 | W | 22 | 2 | Hypertransaminasemia | - | 5 | NA | 41 | 47 | ZnAc | No biochemical remission | 3 |
|  |  | EW71 | M | 36 | 8 | Hypertransaminasemia | - | 5 | NA | 6 | 404 | ZnAc | No biochemical remission | 5 |
| fEW44 | p.G85V /  p.H1069Q | EW77 | W | 29 | 18 | Cirrhosis (F4), steatosis, acute hepatic failure.  Dystonia, psychiatric symptoms | + | 16 | NA | 50 | 533 | D-Pen | Biochemical remission.  Neurological improvement | 7 |
|  |  | EW90 | W | 29 | 27 | Fibrosis (F2-3), steatosis, hypertransaminasemia. Dystonia, instability, dysarthria, dysphagia, psychiatric symptoms | + | 7 | NA | 15 | NA | ZnAc | Biochemical remission.  Neurological improvement | 7 |
| fEW59 | p.M645R /  c.3061-12T>A | EW106 | M | 34 | 34 | Fibrosis (F3-4), steatosis, hypertransaminasemia | - | 3 | 1800 | 5 | 91 | D-Pen | Biochemical remission | 4 |
| fEW66 | c.-447C>T  (homozygous) | EW182^g^ | W | 37 | 3 | Hypertransaminasemia | - | 3 | NA | 5 | NA | ZnAc | Biochemical remission | 3 |
| fEW74 | p.L1120*  (homozygous) | EW210^g^ | W | 40 | NA | Hypertransaminasemia. | - | 5 | NA | 16 | NA | D-Pen | Biochemical remission | 3 |
| fEW90 | p.S1362F /  p.P690L | EW222^g^ | W | 16 | NA | Hypertransaminasemia | - | 5 | NA | 7 | NA | D-Pen | Biochemical remission | 3 |
| fEW102 | p.M645R /  p.H1069Q | EW248^g^ | W | 48 | NA | Cirrhosis, hypertransaminasemia | - | 7 | NA | 20 | NA | D-Pen | Biochemical remission | 4 |

Abbreviations: CP, ceruloplasmin; DG, diagnosis; D-penicillamine, D-Pen; M, Man; KF, Kayser-Fleischer; NA, Not Available; W, Woman; Zinc acetate, ZnAc.

^a^Serum ceruloplasmin levels, expressed in milligrams per deciliter (pathological levels <20 mg/dL). ^b^Hepatic copper levels, expressed in micrograms per gram dry weight (normal levels <50 µg/g dry weight). ^c^Serum copper concentration as micrograms per liter (normal levels 60-120 µg/L). ^d^Urinary daily copper excretion at diagnosis, expressed in µg/24 h. ^e^Leipzig score system, without genetics. ^f^Two patients could not be studied in the Follow-up Cohort. ^g^Seven new patients were recruited and included in the Follow-up Cohort.

**Table S2.** Clinical features of the Validation Cohort.

| Family | *ATP7B* mutations | Patient | Sex | Age (years) | | Presentation | KF  ring | CP^a^ | Copper | | | Treatment | Outcome | Leipzig  Score^e^ |
| --- | --- | --- | --- | --- | --- | --- | --- | --- | --- | --- | --- | --- | --- | --- |
|  |  |  |  | current | DG |  |  |  | Liver^b^ | Serum^c^ | 24h Urine^d^ |  |  |  |
| fMD-332 | p.L708P  (homozygous) | MD-722 | W | 55 | 19 | Cirrhosis, normal transaminase levels.  Dysarthria, seizures | + | 2 | NA | 2 | 160 | ZnAc | No neurological remission. Biochemical remission, ascites | 8 |
| fMD-333 | p.L708P  (homozygous) | MD-723 | M | 54 | 17 | Cirrhosis.  Dystonia, hypertransaminasemia | - | 6 | NA | 30 | 195 | D-Pen | Biochemical remission | 6 |
| fMD-334 | p.L708P /  p.M769V | MD-724 | W | 50 | 12 | Fibrosis (F1).  Hypertransaminasemia | - | 6 | 400 | 17 | 108 | D-Pen | Biochemical remission | 5 |
| fMD-335 | p.L708P  (homozygous) | MD-725 | W | 45 | 17 | Cirrhosis, normal transaminase levels | + | 4 | NA | 46 | 290 | ZnAc | Compensated liver cirrhosis. Biochemical remission | 6 |
| fMD-336 | p.L708P /  p.M645R | MD-726 | M | 48 | 14 | Chronic active hepatitis, hypertransaminasemia | - | 1 | 300 | 11 | 1088 | D-Pen | No biochemical remission | 6 |
| fEW-52 | c.-436_-422del15/  c.3061-12T>A | EW-124 | M | 56 | 10 | Cirrhosis,  hypertransaminasemia | - | 5 | 1013 | 59 | 65 | Trientine | Biochemical remission | 5 |
| fEW-93 | p.R778W /  p.N1270S | PWD-07 | M | 57 | 12 | Hypertransaminasemia | - | 3 | NA | 16 | 71 | D-Pen | Biochemical remission | 3 |
| fMD-337 | p.L708P  (homozygous) | MD-727 | M | 56 | 15 | Cirrhosis, normal transaminase levels.  Dystonia, tremor | + | 3 | 336 | 87 | 56 | D-Pen | Neurological improvement. | 10 |
| fMD-338 | p.L708P /  p.M645R | MD-728 | M | 35 | 9 | Fibrosis (F2),  hypertransaminasemia, hypercholesterolemia, hepatomegaly (1 cm) | - | 5.48 | 119 | 21 | 32 | ZnAc | Biochemical remission | 3 |
| fEW-43 | c.-436_-422del15  (homozygous) | EW-102 | W | 30 | 6 | Steatosis,  hypertransaminasemia | - | 3.83 | 1200 | 14 | 20 | ZnAc | Biochemical remission | 4 |
| fMD-339 | p.L708P /  p.A1135Nfs | MD-729 | M | 35 | 8 | Chronic active hepatitis, hypertransaminasemia, hepatomegaly (1 cm) | - | 3.7 | NA | 14 | 96 | D-Pen | Biochemical remission | 3 |
| fMD-340 | p.L708P  (homozygous) | MD-730 | M | 32 | 12 | Cirrhosis,  hepatomegaly (0.5 cm),  hypertransaminasemia,  maduration delay with family history | - | 7.78 | 874 | 38 | 52 | D-Pen | No biochemical remission | 4 |
| fEW-56 | p.L708P /  p.N1270S | EW-103 | W | 63 | 12 | Cirrhosis, hypertransaminasemia.  Gait problems, depression | + | 5 | NA | 40 | 50 | ZnAc | Neurological improvement. Biochemical remission | 6 |
| fEW-52 | c.-436_-422del15/  c.3061-12T>A | EW-96 | W | 75 | 35 | Asymptomatic | + | 5 | 598 | 24 | 110 | D-Pen | Alive without complications | 8 |
| fEW-57 | p.L708P /  p.M645R | EW-104 | W | 69 | 38 | Asymptomatic | + | 3 | NA | 15 | 1850 | D-Pen | Alive without complications | 6 |
| fMD-341 | p.L708P  (homozygous) | MD-731 | W | 44 | 7 | Hypertransaminasemia, steatosis | - | 9 | NA | 40 | 169 | ZnAc | No biochemical remission | 3 |
| fMD-342 | p.L708P  (homozygous) | MD-732 | M | 52 | 13 | Cognitive problems | + | 9 | NA | NA | 580 | D-Pen | Neurological improvement | 6 |
| fEW-77 | c.-436_-422del15/  p.Ala1135fs | EW-199 | M | 20 | 15 | Hypertransaminasemia | - | 2 | NA | 20 | 61 | D-Pen + ZnAc | Biochemical remission | 4 |
| fEW-19 | c.4124+5G>A/  p.M645R | EW-29 | M | 23 | 6 | Hypertransaminasemia | - | 2 | 864 | 23 | 123 | D-Pen + ZnAc | Biochemical remission | 4 |
| fEW-52 | c.-436_-422del15/  c.3061-12T>A | EW-125 | W | 74 | NA | Asymptomatic. Chronic persistent hepatitis | - | 6 | NA | 20 | 62 | D-Pen | Alive without complications | 6 |
| fMD-343 | p.L708P /  p.G1266W | MD-733 | M | 35 | 9 | Hypertransaminasemia, hepatomegaly (3cm) | - | 3.18 | NA | 40 | 168 | ZnAc | Biochemical remission | 3 |
| fEW-95 | p.E1064K /  p.V1216M + p.P1379S | EW-234 | W | NA | 22 | Hypertransaminasemia | - | 3 | NA | 14 | >500 | D-Pen | Initial biochemical remission until stopping treatment | 4 |

Abbreviations: CP, ceruloplasmin; DG, diagnosis; D-penicillamine, D-Pen; M, Man; KF, Kayser-Fleischer; NA, Not Available; W, Woman; Zinc acetate, ZnAc.

^a^Serum ceruloplasmin levels, expressed in milligrams per deciliter (pathological levels <20 mg/dL). ^b^Hepatic copper levels, expressed in micrograms per gram dry weight (normal levels <50 µg/g dry weight). ^c^Serum copper concentration as micrograms per liter (normal levels 60-120 µg/L). ^d^Urinary daily copper excretion at diagnosis, expressed in µg/24 h. ^e^Leipzig score system, without genetics.

**Table S3**. Demographic data and BMI (Body Mass Index) of the control cohorts.

|  | 1^st^ Control Cohort^#^  for discovery cohort | 2^nd^ Control Cohort^#^  for validation cohort | 3^rd^ Control Cohort^#^  for follow-up cohort | *P*-value |
| --- | --- | --- | --- | --- |
| *n* | 20 | 21 | 25 |  |
| Females | 11 | 10 | 13 |  |
| Males | 9 | 11 | 12 |  |
| Age | 35.60 ± 8.33 (20-56) | 43.14 ± 15.28 (15-70) | 38 ± 9.15 (23-56) | ns |
| BMI | 24.13 ± 3.75 | 26.44 ± 3.50 | NA | ns |
| BMI, Body Mass Index. #For each item, the absolute value or the mean ± standard deviation and the range are indicated in parentheses, as appropriate. ns: not significant; ***P*<0.01. | | | | |

**Table S4**. TaqMan advanced probes used to amplify circulating miRNAs by quantitative PCR for validation.

| **miRNA** | **Reference** | **Sequence** |
| --- | --- | --- |
| hsa-miR-122-5p | 477855_mir | UGGAGUGUGACAAUGGUGUUUG |
| hsa-miR-192-5p | 478262_mir | CUGACCUAUGAAUUGACAGCC |
| hsa-miR-885-5p | 478207_mir | UCCAUUACACUACCCUGCCUCU |
| hsa-miR-885-3p | 479188_mir | AGGCAGCGGGGUGUAGUGGAUA |
| hsa-miR-485-3p | 478125_mir | GUCAUACACGGCUCUCCUCUCU |
| hsa-miR-340-3p | 478041_mir | UCCGUCUCAGUUACUUUAUAGC |
| hsa-miR-193b-5p | 478742_mir | CGGGGUUUUGAGGGCGAGAUGA |
| hsa-miR-455-5p | 478113_mir | UAUGUGCCUUUGGACUACAUCG |
| hsa-miR-16-5p | 477860_mir | UAGCAGCACGUAAAUAUUGGCG |
| hsa-miR-484 | 478308_mir | UCAGGCUCAGUCCCCUCCCGAU |
| hsa-miR-191-5p | 477952_mir | CAACGGAAUCCCAAAAGCAGCUG |
| cel-miR-39 | 478293_mir | UCACCGGGUGUAAAUCAGCUUG |

**Table S5**. Clinical and demographic data of the patients’ cohorts.

|  | Discovery Cohort^#^ | Validation Cohort^#^ | *P*-value |
| --- | --- | --- | --- |
| *n* | 20 | 21 |  |
| Demographic data | | | |
| Females | 11 | 10 |  |
| Males | 9 | 11 |  |
| Age | 36.20 ± 9.10 (21-56) | 43.30 ± 15.23 (15-70) | ns |
| Age since diagnosis (years) | 18.45 ± 9.27 | 27.25 ± 10.28 | ** |
| Disease presentation | | | |
| Age at diagnosis (years old) | 16 ± 12.40 (2-48) | 15.60 ± 9.94 (6-38) | ns |
| Hepatic | 10 | 9 |  |
| Neurologic | 4 | 7 |  |
| Mixed | 2 | - |  |
| Asymptomatic | 4 | 5 |  |
| Biochemical parameters | | | |
| Cholesterol (mg/dL) | 178.60 ± 44.14 | 181.70 ± 31.22 | ns |
| Triglycerides (mg/dL) | 129.7 ± 53.31 | 129.6 ± 62.94 | ns |
| Aspartate aminotransferase  (AST) (U/L) | 39.16 ± 25.25 | 32.32 ± 11.37 | ns |
| Alanine aminotransferase  (ALT) (U/L) | 63.80 ± 74.92 | 45.80 ± 29.01 | ns |
| Gamma-glutamyl transferase  (GGT) (U/L) | 42.35 ± 24.38 | 43.90 ± 41.43 | ns |
| Alkaline phosphatase (U/L) | 84.55 ± 35.64 | 93.95 ± 28.70 | ns |
| Total bilirubin (mg/dL) | 0.66 ± 0.35 | 0.99 ± 0.81 | ns |
| # For each item, the absolute value or the mean ± standard deviation and the range are indicated in parentheses, as appropriate. ns: not significant; ***P*<0.01. | | | |

|  | 1^st^ Control Cohort^#^  for discovery cohort | 2^nd^ Control Cohort^#^  for validation cohort | 3^rd^ Control Cohort^#^  for follow-up cohort | *P*-value |
| --- | --- | --- | --- | --- |
| *n* | 20 | 21 | 25 |  |
| Females | 11 | 10 | 13 |  |
| Males | 9 | 11 | 12 |  |
| Age | 35.60 ± 8.33 (20-56) | 43.14 ± 15.28 (15-70) | 38 ± 9.15 (23-56) | ns |
| BMI | 24.13 ± 3.75 | 26.44 ± 3.50 | NA | ns |
| BMI, Body Mass Index. #For each item, the absolute value or the mean ± standard deviation and the range are indicated in parentheses, as appropriate. ns: not significant; ***P*<0.01. | | | | |

| **Table S6.**  Significantly deregulated mature miRNAs using the QLF (Quasi-Likelihood F Test) and the LRT (Likelihood Ratio Test) methods. | | | | | | | | | | | | | | | | |
| --- | --- | --- | --- | --- | --- | --- | --- | --- | --- | --- | --- | --- | --- | --- | --- | --- |
|  | **LRT design 1** | | | | **QLF design 1** | | | | **LRT design 2** | | | | **QLF design 2** | | | |
|  | **log2FC** | ***P*-value*** | **FDR** | **log2FC** | | ***P*-value*** | **FDR** | **log2FC** | | ***P*-value*** | **FDR** | **log2FC** | | ***P*-value*** | **FDR** |  |
| **hsa-miR-122-5p** | **3.38** | **1.48E-13** | **1.83E-10** | **3.38** | | **1.02E-08** | **1.23E-05** | **2.77** | | **2.85E-10** | **5.22E-07** | **2.77** | | **2.39E-06** | **1.72E-03** |  |
| hsa-miR-122b-3p | 3.38 | 1.70E-13 | 1.83E-10 | 3.38 | | 1.14E-08 | 1.23E-05 | 2.77 | | 4.84E-10 | 5.22E-07 | 2.77 | | 3.44E-06 | 1.86E-03 |  |
| **hsa-miR-193b-5p** | **2.64** | **1.50E-10** | **1.08E-07** | **2.64** | | **1.76E-07** | **1.27E-04** | **1.91** | | **4.66E-09** | **2.52E-06** | **1.92** | | **1,83E-07** | **1.97E-04** |  |
| **hsa-miR-885-3p** | **3.25** | **4.63E-10** | **2.50E-07** | **3.23** | | **2.38E-07** | **1.28E-04** | **2.67** | | **8.42E-08** | **3.64E-05** | **2.67** | | **7.91E-06** | **2.42E-03** |  |
| **hsa-miR-192-5p** | **1.76** | **1.07E-07** | **3.84E-05** | **1.76** | | **2.14E-06** | **6.59E-04** | **1.33** | | **1.16E-05** | **2.08E-03** | **1.33** | | **6.47E-05** | **1.16E-02** |  |
| **hsa-miR-885-5p** | **2.46** | **1.54E-07** | **4.75E-05** | **2.47** | | **4.81E-06** | **1.15E-03** | **2.19** | | **8.84E-06** | **1.73E-03** | **2.17** | | **1.50E-04** | **1.94E-02** |  |
| hsa-miR-125b-5p | 1.33 | 2.19E-07 | 5.91E-05 | 1.33 | | 1.53E-06 | 5.50E-04 | 1.07 | | 4.30E-05 | 5.60E-03 | 1.07 | | 1.02E-04 | 1.57E-02 |  |
| **hsa-miR-455-5p** | **2.03** | **6.21E-07** | **1.34E-04** | **2.02** | | **1.08E-05** | **2.11E-03** | **1.75** | | **4.00E-07** | **1.44E-04** | **1.75** | | **8.42E-06** | **2.42E-03** |  |
| hsa-miR-99a-5p | 1.31 | 1.99E-06 | 3.90E-04 | 1.31 | | 1.45E-05 | 2.38E-03 | 1.05 | | 1.11E-04 | 1.12E-02 | 1.05 | | 3.50E-04 | 2.90E-02 |  |
| hsa-miR-200b-3p | 1.31 | 2.45E-06 | 4.12E-04 | 1.31 | | 3.54E-06 | 9.56E-04 | 1.18 | | 6.36E-06 | 1.38E-03 | 1.18 | | 8.96E-06 | 2.42E-03 |  |
| **hsa-miR-485-3p** | **1.96** | **2.78E-06** | **4.12E-04** | **1.96** | | **4.13E-05** | **5.24E-03** | **1.76** | | **2.24E-09** | **1.61E-06** | **1.76** | | **1.79E-08** | **3.86E-05** |  |
| hsa-miR-200a-3p | 1.33 | 3.03E-06 | 4.12E-04 | 1.33 | | 6.84E-06 | 1.48E-03 | 1.10 | | 1.23E-04 | 1.12E-02 | 1.10 | | 1.53E-04 | 1.94E-02 |  |
| hsa-miR-122b-5p | 2.23 | 3.05E-06 | 4.12E-04 | 2.23 | | 1.54E-05 | 2.38E-03 | 2.09 | | 2.10E-05 | 3.09E-03 | 2.09 | | 2.22E-04 | 2.28E-02 |  |
| hsa-miR-193a-5p | 1.42 | 6.06E-06 | 7.34E-04 | 1.42 | | 2.69E-05 | 3.77E-03 | 1.11 | | 1.52E-04 | 1.26E-02 | 1.11 | | 3.36E-04 | 2.90E-02 |  |
| hsa-miR-30a-5p | 1.17 | 6.12E-06 | 7.34E-04 | 1.17 | | 2.79E-05 | 3.77E-03 | 1.00 | | 9.35E-05 | 1.06E-02 | 1.00 | | 2.49E-04 | 2.34E-02 |  |
| **hsa-miR-340-3p** | **1.43** | **2.31E-05** | **2.49E-03** | **1,43** | | **1.05E-04** | **1.08E-02** | **1.43** | | **1.15E-06** | **3.54E-04** | **1.43** | | **4.81E-06** | **2.07E-03** |  |
| hsa-miR-511-5p | 1.09 | 4.36E-05 | 4.48E-03 | 1.09 | | 4.86E-05 | 5.52E-03 | 0.97 | | 2.08E-04 | 1.55E-02 | 0.97 | | 2.09E-04 | 2.25E-02 |  |
| hsa-miR-23b-3p | 1.18 | 1.85E-04 | 1.59E-02 | 1.18 | | 3.68E-04 | 3.46E-02 | 1.09 | | 1.63E-04 | 1.30E-02 | 1.10 | | 1.91E-04 | 2.17E-02 |  |
| **P*-value adjusted using the Benjamini-Hochberg method to reduce the False Discovery Rate (FDR); log_2_FC: logarithm to base 2 of the fold change. Eight miRNAs were first selected (in bold), and five miRNAs were validated (in blue). | | | | | | | | | | | | | | | |  |
